# Supplementary material for: IL-10 Enhances the Inhibitory Effect of Adipose-Derived Stromal Cells on Insulin Resistance/Liver Gluconeogenesis by Treg Cell Induction
Source: Int J Mol Sci. 2024 Jul 25;25(15):8088. doi: 10.3390/ijms25158088 (PMC11311376; doi:10.3390/ijms25158088)
Supplement: Supplementary file 1 [file ijms-25-08088-s001.zip › ijms-3075253-supplementary.pdf]

IL-10-treated adipose-derived stromal cells decrease insulin resistance/liver gluconeogenesis through Tregs in a type 2 diabetes mellitus mouse model

Hsiao-Chi Lai[1,2], Pei-Hsuan Chen[1,2], Chia-Hua Tang[1], Lee-Wei Chen[1,2,3*]

Supplemental Figures

Supplemental Figure S1: SVFs were harvested from the adipose tissue of *Lepr*$^{db/db}$ mice and treated with PBS, 10 ng IL-10, or 100 ng IL-10 for 3.5 h followed by western blotting analysis of IL-6, pmTOR, pJNK, and pNFκB protein expression. Representative images and statistical analysis are presented in Figure 2A.

protein marker ↓

p-mTOR->

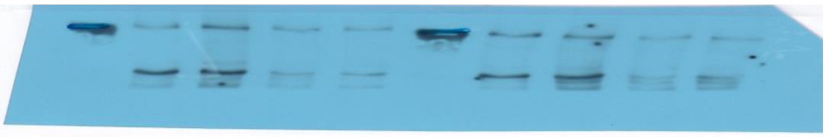

protein marker ↓

IL-6->

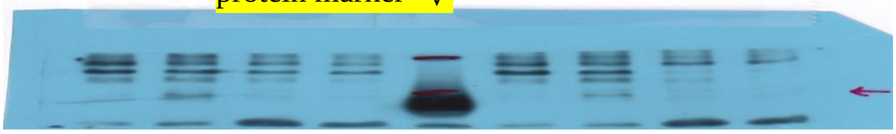

protein marker ↓

IL-6->

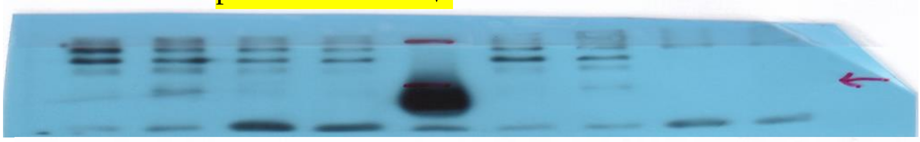

protein marker ↓

IL-6->

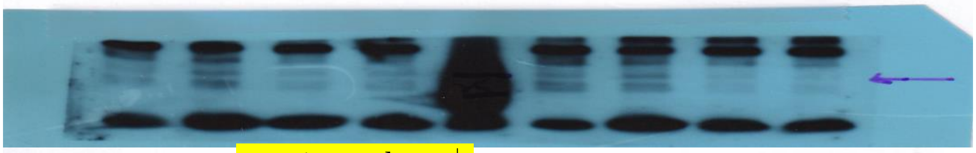

protein marker ↓

p-JNK->

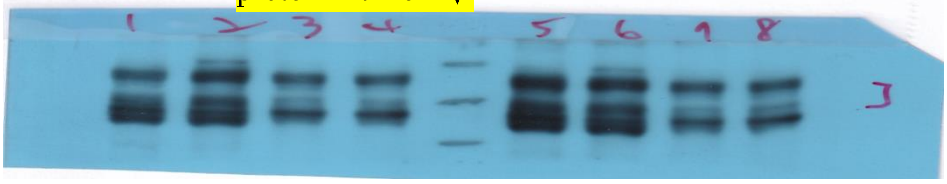

p-JNK->

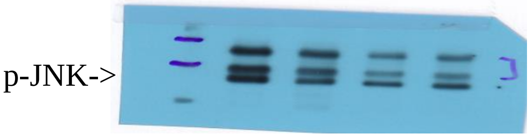

protein marker ↓

p-NFkB->

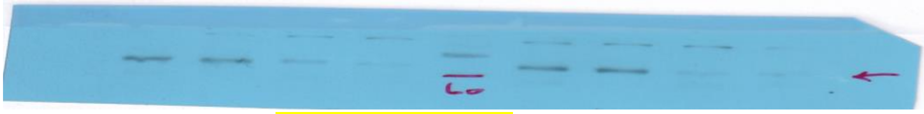

protein marker ↓

NF-kB->

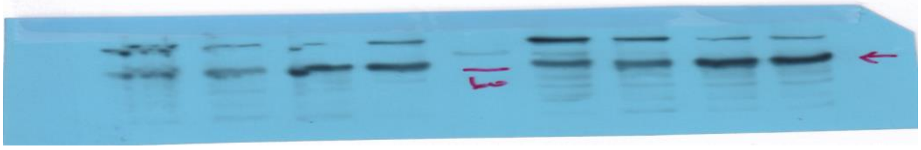

protein marker ↓

B-actin

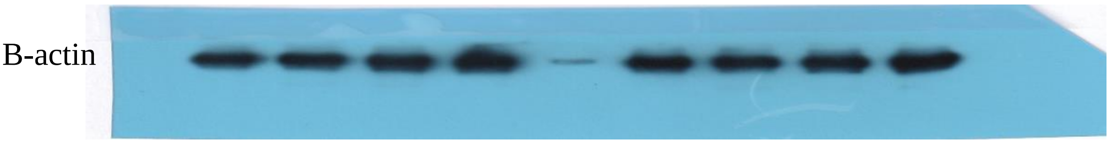

PBS PBS 10 ng IL-10 100 ng IL-10 PBS PBS 10 ng IL-10 100 ng IL-10

Lepr$^{+/+}$ *Lepr*$^{db/db}$ *Lepr*$^{+/+}$ *Lepr*$^{db/db}$

Supplemental Figure S2: Injection of IL-10-treated SVFs increase Akt, ERK, and STAT3 protein activation and attenuate glucose intolerance in *Lepr*$^{db/db}$ mice. Uncropped Western blot images of pAkt, Akt, pERK, STAT3, pSTAT3, and ERK of liver. Representative images and statistical analysis are presented in Figure 7A.

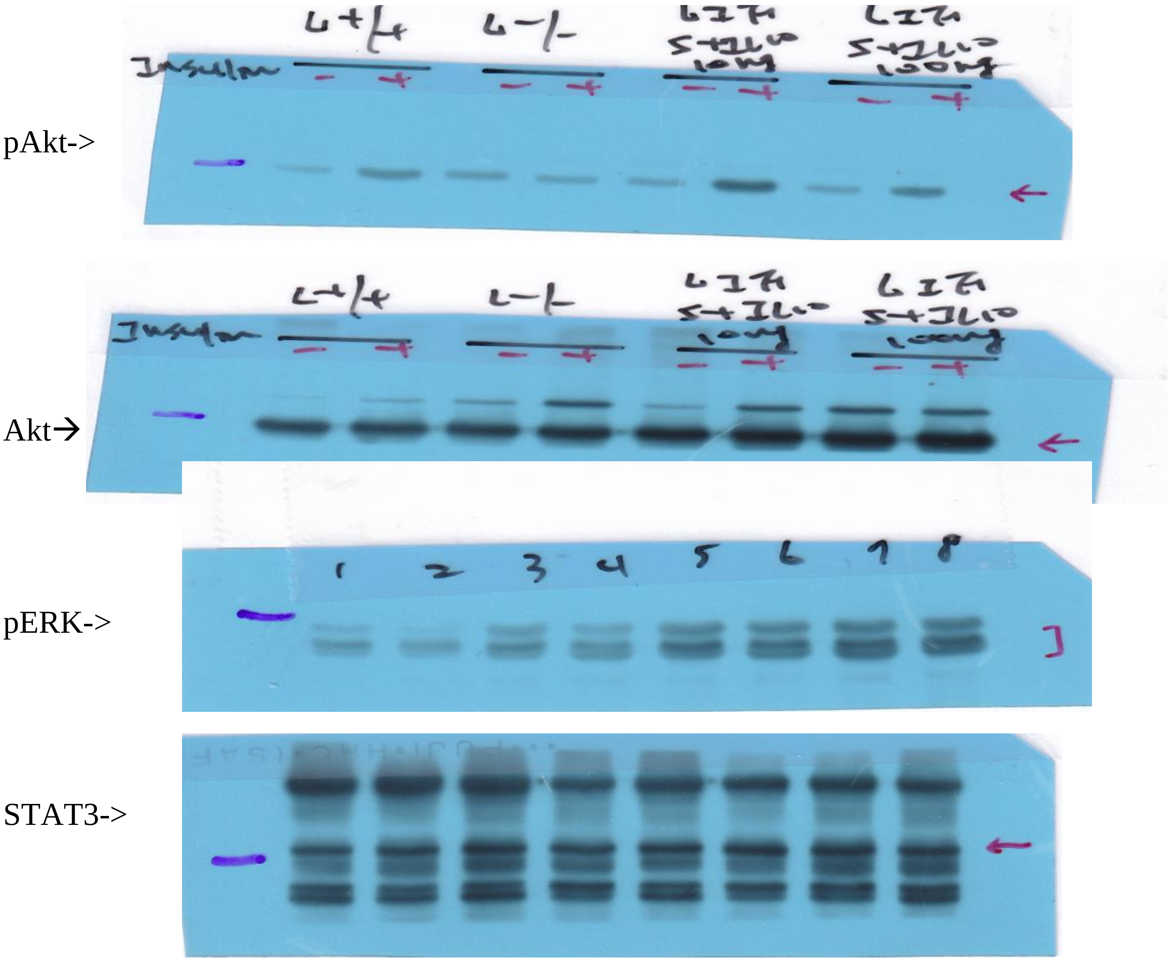

pAkt->

Akt→

pERK->

STAT3->

p-STAT3

b-actin->

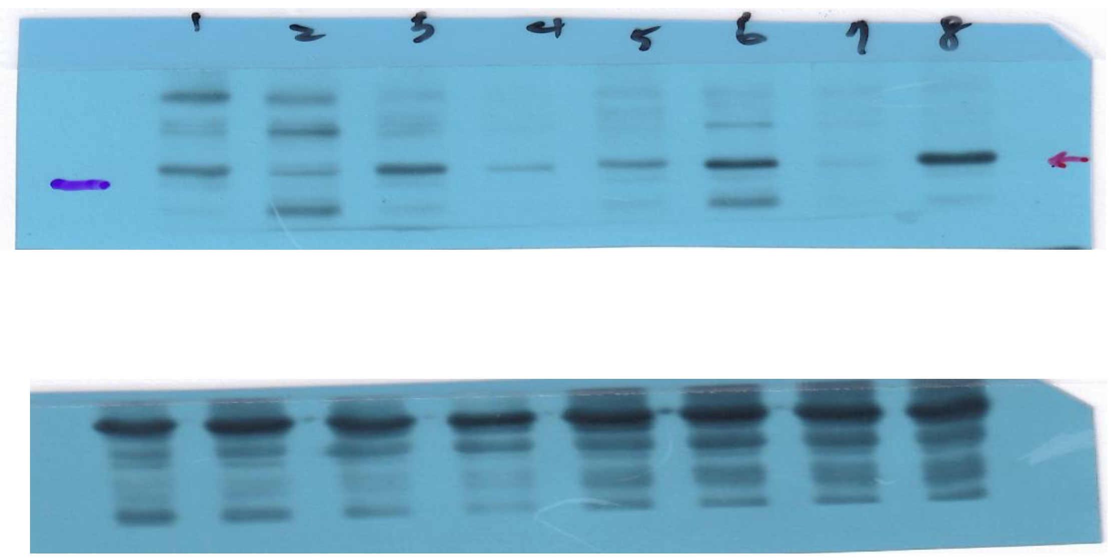

	Lepr$^{+/+}$	*Lepr*$^{db/db}$	*Lepr*$^{db/db}$ SVF IL-10 10 ng	*Lepr*$^{db/db}$ SVF IL-10 100 ng
**Insulin**	**-** **+**	**-** **+**	**-** **+**	**-** **+**
